# Dynamic diffraction effects and coherent breathing oscillations in ultrafast electron diffraction in layered 1*T*-TaSeTe

**DOI:** 10.1063/1.4979643

**Published:** 2017-03-30

**Authors:** Linlin Wei, Shuaishuai Sun, Cong Guo, Zhongwen Li, Kai Sun, Yu Liu, Wenjian Lu, Yuping Sun, Huanfang Tian, Huaixin Yang, Jianqi Li

**Affiliations:** 1 Beijing National Laboratory for Condensed Matter Physics, Institute of Physics, Chinese Academy of Sciences, Beijing 100190, China; 2 School of Physical Sciences, University of Chinese Academy of Sciences, Beijing 100049, China; 3 Key Laboratory of Materials Physics, Institute of Solid State Physics, Chinese Academy of Sciences, Hefei 230031, China; 4 High Magnetic Laboratory, Chinese Academy of Sciences, Hefei 230031, China; 5 Collaborative Innovation Center of Advanced Microstructures, Nanjing University, Nanjing 210093, China; 6 Collaborative Innovation Center of Quantum Matter, Beijing 100084, China

## Abstract

Anisotropic lattice movements due to the difference between intralayer and interlayer bonding are observed in the layered transition-metal dichalcogenide 1*T*-TaSeTe following femtosecond laser pulse excitation. Our ultrafast electron diffraction investigations using 4D-transmission electron microscopy (4D-TEM) clearly reveal that the intensity of Bragg reflection spots often changes remarkably due to the dynamic diffraction effects and anisotropic lattice movement. Importantly, the temporal diffracted intensity from a specific crystallographic plane depends on the deviation parameter s, which is commonly used in the theoretical study of diffraction intensity. Herein, we report on lattice thermalization and structural oscillations in layered 1T-TaSeTe, analyzed by dynamic diffraction theory. Ultrafast alterations of satellite spots arising from the charge density wave in the present system are also briefly discussed.

## I. INTRODUCTION

The novel physical properties of two-dimensional (2D) materials such as graphene and layered MX_2_ transition-metal dichalcogenides (TMDs, M=Mo, W, V, Nb, Ta, Ti, Zr, Hf, or Re and X=Se, S, or Te) have received much interest. They are regarded as having potential to revolutionize many application fields, such as electronics, energy storage, and optics.^1–3^ TMDs are composed of X-M-X layers, and these stacked layers are weakly bound together by the van der Waals force. The M atoms in each sheet occupy the center of common octahedral edges formed by covalently bonded X atoms. Variations in stacking sequences of MX_2_ layers, together with atomic coordination, lead to structural polymorphism.^4–7^ In addition, layered TMDs have been extensively studied due to their multiple electronic features, such as charge density waves (CDWs) and superconductivity.^4–7^ Recently, ultrafast X-ray diffraction (UXRD)^8–10^ and ultrafast electron diffraction (UED)^9–12^ have provided insight into the atomic structural dynamics in theses types of 2D materials, and most UXRD and UED research on TMDs is focused on photo-induced processes in CDW melting^13–17^ and multi-phase transition.^18–20^ In particular, the temporal changes of the diffraction intensity and satellite spots within the a*-b* plane of reciprocal space have been extensively investigated. On the other hand, it is expected that the lattice dynamics in other crystallographic planes can provide more structural information for understanding the structural evolutions.

In the study of ultrafast diffraction, it has been well demonstrated that the diffraction intensity change of a specific Bragg spot often exhibits an anomaly arising from the Debye-Waller effect^21,22^ and coherent oscillations.^23–28^ In addition to the intensity oscillations induced by coherent optical phonons (bismuth^29–31^ and graphite^27^), it is believed that the anomalous intensity change originates from zone-axis tilting caused by surface deformation or anisotropic lattice expansion.^23^ Moreover, the thermal expansion coefficient of the c-axis in such layered-structure materials is often much larger than that in the a-b plane, and this anisotropic feature commonly yields a large-amplitude breathing standing-wave and anisotropic periodic oscillation in lattice parameters.^23^ Observations of such a breathing mode in thin film samples have also been extensively reported, including metal (aluminum,^32^ gold,^33^ and silver^34^), semimetal (bismuth^25^), and semiconductor (silicon^24^) films.

In this paper, we report on the ultrafast dynamic evolutions observed in 1*T*-TaSeTe, including the suppression of the satellite spots corresponding to the CDW modulation, the Debye-Waller effect associated with lattice thermalization, and oscillatory evolutions of lattice expansion and diffraction intensity. Based on the crystal dynamic diffraction theory, the essential nature of temporal diffraction intensity alterations has been well interpreted using the Debye-Waller effect together with the plane tilting caused by anisotropic lattice expansion.

## II. EXPERIMENTAL METHOD

A 1*T*-TaSeTe crystalline sample is a doped CDW material which shows a superconducting transition temperature Tc = 0.7 K; this material has a hexagonal unit cell with lattice parameters of a = b = 0.3507 nm and c = 0.6337 nm.^35^ Our recent transmission electron microscopy (TEM) investigations show that the incommensurate CDW state in 1*T*-TaSeTe could result in visible periodic lattice distortion (PLD) similar to that discussed with regard to TaTe_2_.^36^ *In situ* heating TEM observations demonstrate that the intensities of the CDW satellite spots decrease progressively with the increase in temperature, and the spots finally disappear above 900 K, as shown in Fig. S1 (supplementary material).

**FIG. 1.**
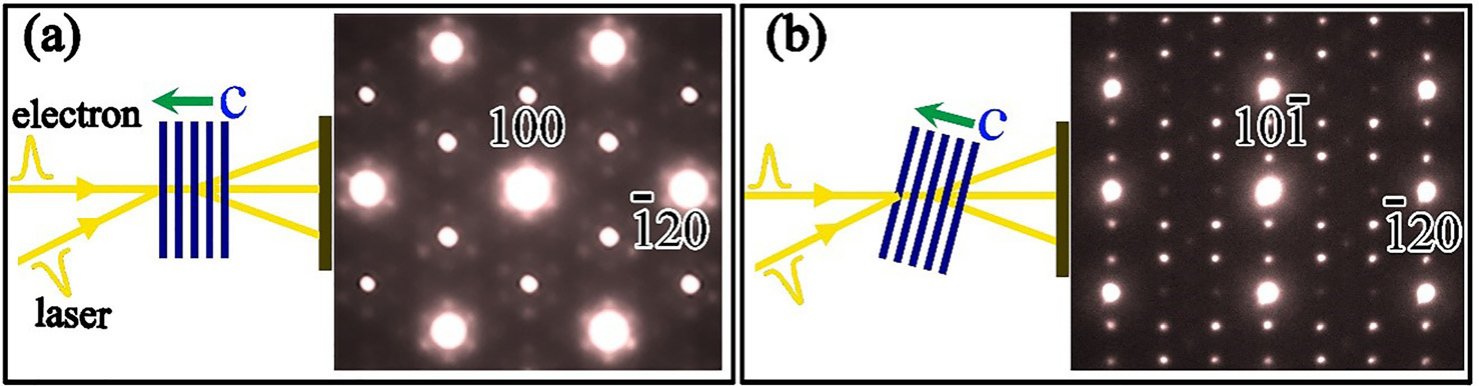
Electron diffraction patterns taken along the [001] (a) and [212] (b) zone axis directions for 1*T*-TaSeTe and schematic diagrams clearly illustrating the crystal orientation relative to the electron beam and the pumping laser.

The single-crystal specimens for ultrafast electron diffraction experiments were exfoliated to a thickness of approximately 60 nm, and the sample slices were mounted on well-aligned multi-walled carbon nanotube bunches, which were textured and arranged in a decussate pattern on TEM Cu-grids for good heat dissipation. In Fig. S2 (supplementary material), we show typical bright-field TEM images of 1*T-*TaSeTe crystals mounted on a TEM grid, and the crystalline samples used for our measurements generally have a lateral size of 5–10 *μ*m. The thin 1*T*-TaSeTe crystals often show their a-b plane perpendicular to the electron beam in 4D-TEM, as shown in Fig. 1(a). Therefore, the in-plane structural dynamic features can usually be observed. We can also tilt the sample to other directions to observe the lattice motions in relevant crystal planes. For instance, we tilt the sample with an angle of 25° to the [212] zone axis direction as shown in Fig. 1(b), in which reflection spots from the a*-b* plane of reciprocal space (hk0), CDW modulations, and reflection spots containing c* components (out of plane stacking direction) can be well observed.

The ultrafast electron diffraction measurements were performed using a home-made ultrafast transmission electron microscopy (UTEM) based on a JEOL-2000EX^37^ operated at a voltage of 160 kV. The LaB_6_ photocathode was driven by 300 fs laser pulses (wavelength: 347 nm and repetition rate: 100 kHz). The sample was pumped at room temperature by another fs laser to initiate ultrafast structural dynamics (wavelength: 520 nm). The laser spot was about 100 *μ*m in diameter. The electron beam was spread to 20 *μ*m, and the selected area aperture diameter was 5 *μ*m in the experiments. We improved the temporal resolution by lowering the probe-laser power applied to obtain photoemission to reduce space-charge effects in the photoelectron propagation process. Each electron pulse contains approximately 60 electrons, and the pulse duration is estimated to be around 1ps (FWHM) at the sample position. We obtained a series of diffraction patterns at a laser fluence of 7 mJ/cm^2^, and each diffraction pattern for the ultrafast process was obtained at an exposure time of 30 s.

## III. RESULTS AND DISCUSSION

**FIG. 2.**
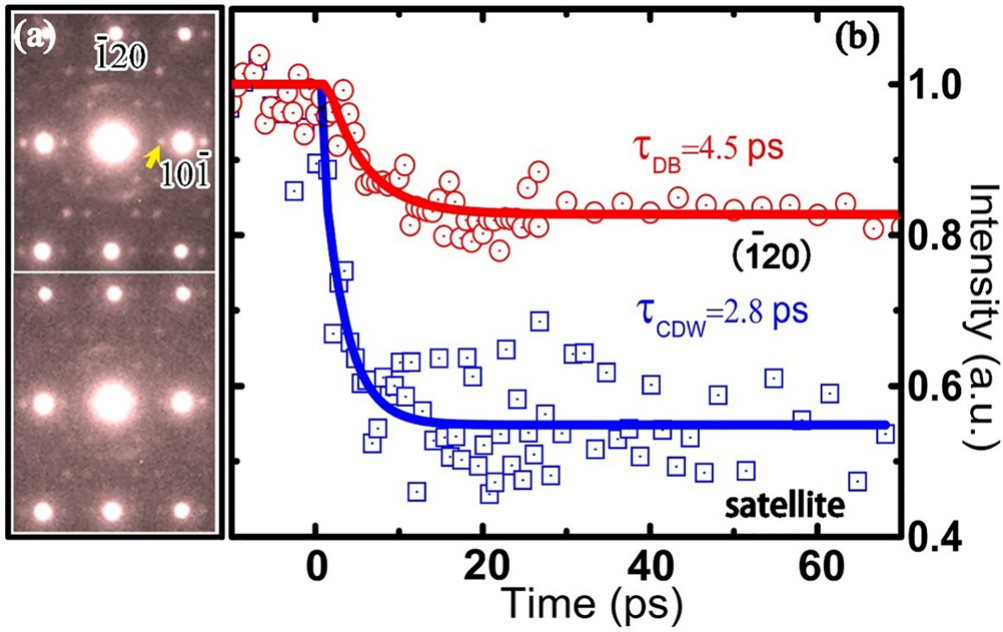
(a) Electron diffraction patterns for time delays of −5 ps (the upper frame) and 10 ps (the lower frame). (b) The intensity evolution of the (1-20) Bragg peak (red circles) and the indicated CDW satellite spot (blue squares), demonstrating the Debye-Waller effect and the suppression of CDW modulation of 1*T*-TaSeTe after laser excitation; the time constants for fitting the experimental data are set as 4.5 ps and 2.8 ps, respectively.

First, we discuss the very visible suppression of CDW satellites and the rapid decrease in the Bragg spot intensity after ultrafast laser excitation. As shown in Fig. 2(a), the temporal diffraction patterns were observed along the [212] zone; the reflection spots that reveal only in-plane structural information and the spots that show out-of-plane components can both be observed in these diffraction patterns. The upper diffraction frame was obtained at a negative delay time of −5 ps, and the lower diffraction pattern was obtained at 10 ps after laser excitation. These experimental results clearly demonstrate that the intensity of satellites related to CDW modulation (one satellite is indicated by the arrow in Fig. 2(a)) was suppressed by the laser excitation. The extracted CDW satellite intensity data from the experimental results are shown by the blue squares in Fig. 2(b). Fitting the time dependence of the satellites' intensity to a single exponential for the entire 60 ps range yields a time constant of 2.8 ps. The suppression of satellite intensity in this layered CDW system can be attributed to atomic motion resulting from electronic spatial redistribution after laser excitation, similar to phenomena discussed in previous ultrafast electron diffraction experiment reports.^13–15^

When energy transfers from excited electrons to phonons, lattice thermalization occurs on a picosecond time scale and generally results in reduced Bragg peak intensity, i.e., the Debye-Waller effect. The temporal change of the (-120) Bragg spot (an in-plane spot without the c-component), depicted in Fig. 2(b) with red circles, shows a notable decreasing transition, which can be well fitted by a single-exponential decay function as

$$I(t)/I_{0}=\left(1-\beta \right)+\beta \;\text{exp}(-t/\tau_{e-ph}),$$ (1)

where *I(t)* is the intensity of a diffraction spot at a given time *t* after excitation, *I_0_* is the intensity before excitation, *β* = 17% is the diffraction intensity decrease caused by the Debye-Waller effect, and *τ_e-ph_* = 4.5 ps is the time constant of lattice thermalization associated with electron-phonon coupling. The temperature increase of the lattice after laser excitation can be estimated by considering a time-dependent Debye Waller factor^21,22^

$$I\left(t\right)/I_{0}=\text{exp}\left(-\frac{3\mu^{2}h^{2}\Delta T\left(t\right)}{4\pi^{2}Mk_{B}\Theta_{M}^{2}}\right),$$ (2)

where *μ* is the scattering vector, *M* is the average mass, *h* is the Planck constant, *k_B_* is the Boltzmann constant, $\Theta_{M}=\Theta_{D}/\sqrt{p}$^38^ ($\Theta_{D}=147K$^7^ and $\;\Theta_{M\;}$ are the Debye temperatures derived from specific heat and from crystallographic analyses, respectively, and *p* is the number of atoms per lattice point), and *ΔT(t)* is the temperature increase following laser excitation. The 17% intensity suppression of the (-120) Bragg spot corresponds roughly to a 125 K rise of the lattice temperature. These data are consistent with the calculated result of 136 K based on the thermal lattice expansion as discussed below.

As is well known, the Debye Waller equation was initially derived for simple monoatomic cubic lattices; in order to analyze experimental data with regard to the 1*T*-TaSeTe material, we used the derivation of the formula reported in Ref. 21 for polyatomic compounds. Moreover, it was demonstrated in the UED study of the CDW 1*T*-TaS_2_ (Ref. 13) and 4H_b_-TaSe_2_ (Ref. 14) systems that the suppression of PLD in CDW materials could lead to a partial enhancement of the periodicity of the host lattice; because the intensity of the CDW satellite spots in the present system is generally much weaker^13–15^ than that of the main Bragg spots, for the qualitative analysis discussed in this paper, we did not analyze the cooperative changes of intensity recovery that arise from the change in the PLD signal.

**FIG. 3.**
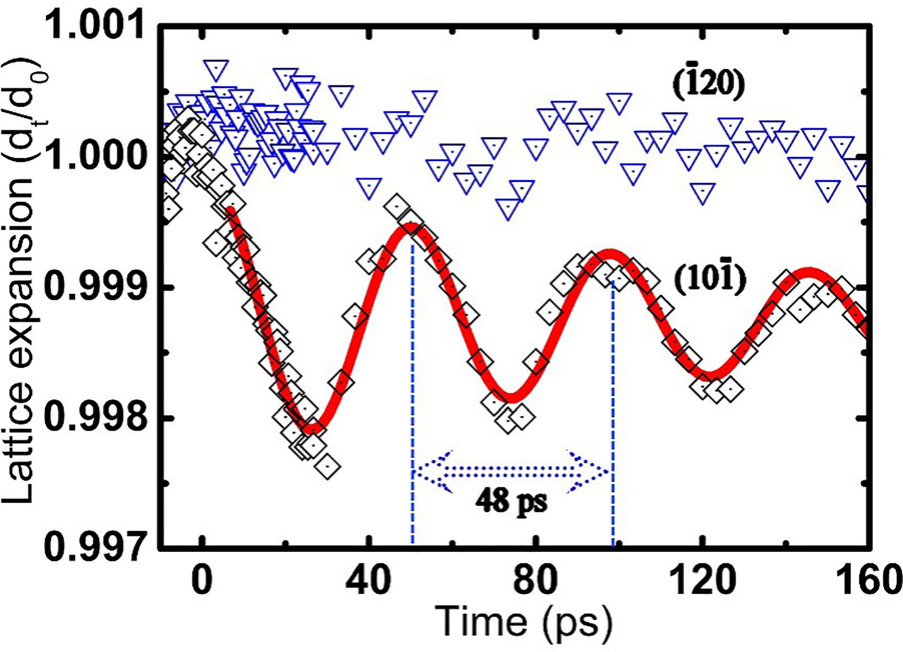
Anisotropic lattice dynamics in 1*T*-TaSeTe. The oscillatory dynamics of [10-1] interplanar space induced by the thermal expansion following photoexcitation can be well fitted with coherent breath oscillation (red solid curve); on the other hand, the [-120] interplanar space shows almost no temporal alteration.

Further detailed analysis of the [212] zone axis experimental data reveals that temporal lattice movements along the (10-1) direction exhibit oscillatory characteristics, whereas lattice expansion within the a-b plane is imperceptible, as shown in Fig. 3. The anisotropic lattice alternation in 1*T*-TaSeTe can be considered to rise from the nature of this system's chemical bonding, i.e., weak van der Waals bonding between layers and strong covalent bonds within the sheets. The remarkable lattice oscillation of (10-1) that occurs after fs-laser excitation with fluence F = 7 mJ/cm^2^ can be well fitted by a damped harmonic phonon vibration.^27,32^ The step function of lattice temperature is adopted to analyze the phonon relaxation and lattice thermalization in the present system, and so the temporal evolution of the inter-planar distance along the [10-1] lattice direction can be described by a decaying oscillatory function:

$$d_{t}=d_{e}+A\;\text{exp}(-t/\tau_{damp})\;\text{cos}(2\pi t/T),$$ (3)

where the fitted curve shows a new thermal equilibrium position with *d_e_* = 0.99875 and an average thermal expansion of about Δ*d* = 1.25‰ after laser excitation, and the damping constant is τ_damp_ = 146 ps. The oscillation can be fitted to a very well-defined period of T = 48 ps. Taking into account the crystal tilting shown in Fig. 1, the expansion of c can be estimated to be Δ*c* = Δ*d*/sin(25°) ≈ 3‰ at the new thermal equilibrium. Considering the thermal expansion coefficient of crystal lattices α(001) = 2.2 × 10^−5^/K, obtained from the *in situ* heating XRD data (Fig. S3 of the supplementary material), we estimate the temperature rise induced by single laser pulses to be 136 K. The lattice vibration displays a nearly cosine-shaped time dependence with a maximum displacement at time zero. Such an oscillatory behavior observed previously on similar time scales in other systems has been attributed to natural acoustic resonances,^24,25,32–34^ also known as the breathing mode acoustic phonon. Fourier transformation of the vibration of the (10–1) Bragg peak yields a single peak centered at 20.8 GHz, which represents the energy of the low-energy acoustic-phonon mode with a period of 48 ps, in agreement with that predicted by the 1D standing-wave condition T = 2 L/v, where L = 60 nm is the thickness of our sample, and the sound velocity in material systems similar to our sample is *v = *2500 m/s.^39,40^

**FIG. 4.**
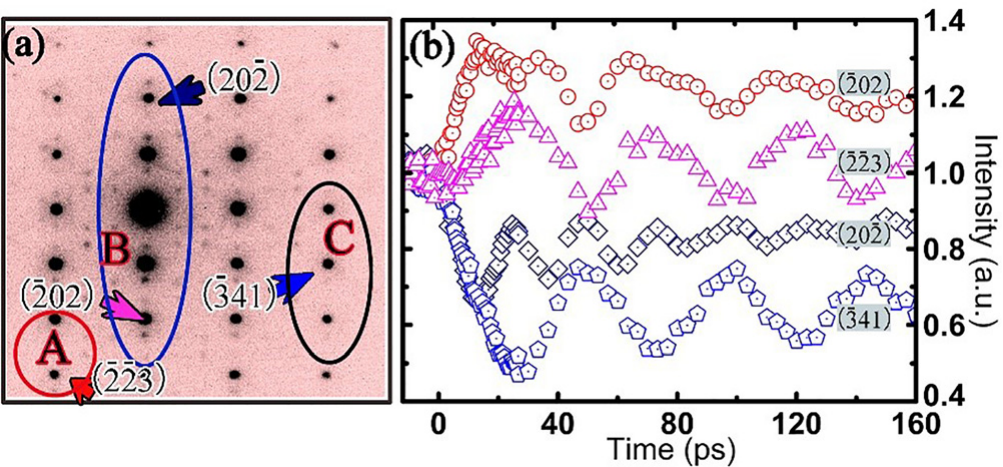
Temporal evolution of the Bragg peak intensity of 1*T*-TaSeTe excited by the fs-laser with a fluence of 7mJ/cm^2^. (a) Electron diffraction pattern slightly off the [212] zone-axis direction. In this pattern, the deviation parameter *s* has been carefully tuned by controlling the crystal orientation, i.e., diffraction spots from the Bragg position have their *s* > 0 in area A, *s* ≅ 0 in area B, and *s* < 0 in area C. (b) Temporal changes of Bragg peak intensities for typical diffraction spots in areas A, B, and C, respectively, clearly illustrating the presence of unusual temporal features for the spots in different areas.

Extensive studies on ultrafast intensity changes of electron diffraction in 1*T*-TaSeTe (especially for the out-of-plane spot with the c component) reveal a rich variety of dynamic structural features. Experimental examinations on a few well-characterized samples demonstrated that the lattice movements and decay of the diffraction intensity clearly depend on the crystallographic orientation and the dynamic diffraction effect. Figure 4(a) shows an ultrafast electron diffraction pattern obtained slightly off the [212] zone axis. In this pattern, the deviation parameter *s* used for the theoretical study of dynamic diffraction effects has been carefully tuned by controlling the crystal orientation, i.e., diffraction spots from the Bragg position have their s > 0 in area A, s ≅ 0 in area B, and s < 0 in area C.

Figure 4(b) presents typical experimental results observed for lattice relaxation in the present system, clearly illustrating the presence of unusual temporal features for different diffraction spots. In area B with *s* ≅ 0, we show the (-202) and (20-2) diffraction curves; it can be clearly seen that the temporal evolution of their intensity is doubly modulated by phonon excitations. The (-2-23) and the (-341) diffraction curves exhibit temporal oscillations in an out-of-phase relation.

**FIG. 5.**
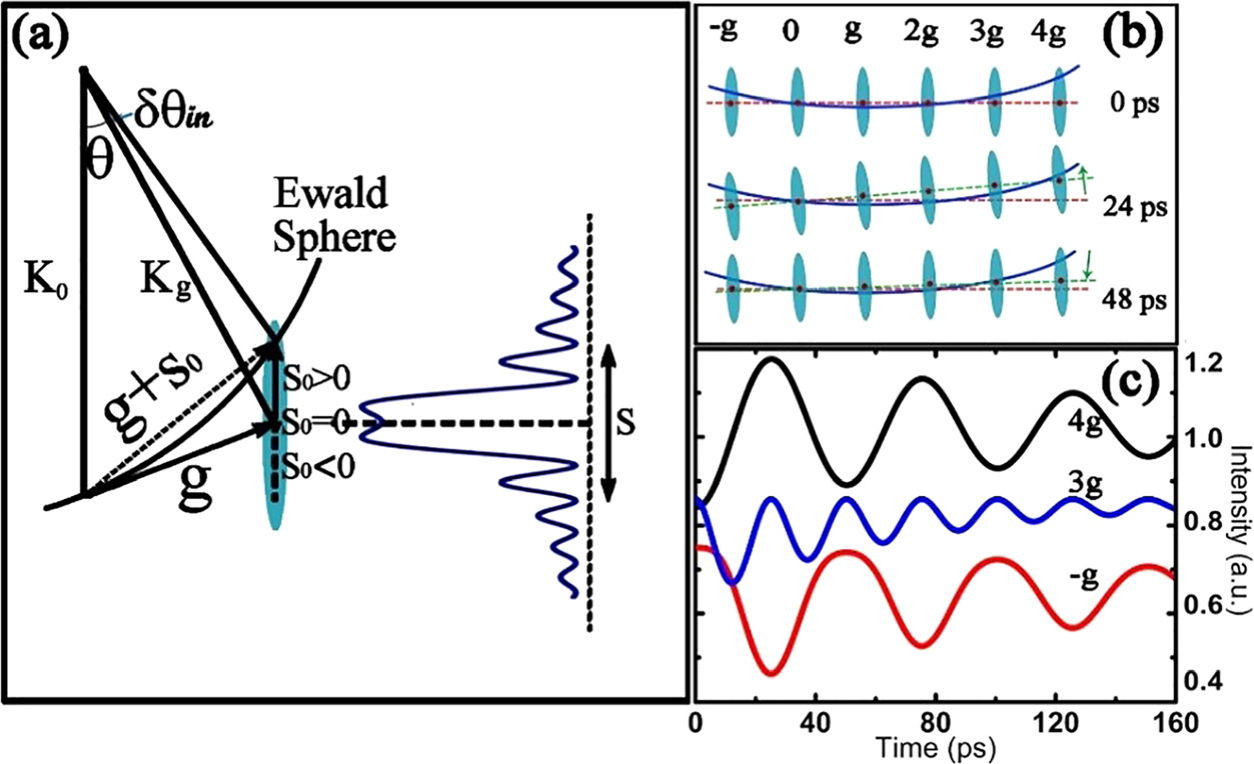
(a) Sketch of the crystal dynamic diffraction theory; the Bragg peak intensity as a function of deviation parameter *s* is shown as a blue curve. (b) Temporal evolution of the intersections of the Ewald sphere (blue curvature line) with the reciprocal rods, which is modulated by the breath oscillation of the thin crystal along the c axis; the red line is the horizontal reference line, and the green line represents the reciprocal plane. The oscillation period is set to be 48 ps, and typical positions for three time delays are displayed. (c) Three simulated results for H = −g, 3g, and 4g, clearly illustrating the appearance of two out-of-phase oscillations (4g, −g) and a half-period oscillation (3g) arising from coupling between the excitation coherent lattice oscillation and the dynamic diffraction effect.

It is commonly noted that the plane tilting arising from in-plane thermal stress^24^ or anisotropic lattice expansion^23^ along the c-axis could yield visible changes in diffraction intensity. We analyzed the diffraction intensity oscillations by performing the Fourier transform, using the same method mentioned in Ref. 24. Remarkably, the two extracted frequency components, 20.8 and 41.6 GHz, correspond, respectively, to oscillation periods of 48 and 24 ps, both of which are believed to correlate essentially with the excitation of the breathing mode. Furthermore, considering the homogeneous excitation of our sample, the only excited coherent phonon mode was the longitudinal coherent acoustic phonon, similar to results observed in bismuth thin film^25^ and graphite.^23,27^ Neither a kinematic diffraction formula nor a rocking curve properly explains the ultrafast alterations of diffraction intensity. An extensive theoretical analysis of multi-beam dynamical diffraction is in progress. In the present case, we qualitatively analyzed the Bragg peak intensity evolutions using the time based two-beam dynamical diffraction theory under the assumption of no absorption.^41,42^ According to the theory, the intensity of the Bragg peak is described as

$$I_{hkl}=\frac{1}{1+{\left(\xi_{g}s\right)}^{2}}{\text{sin}}^{2}\left(\frac{\pi l}{\xi_{g}}\sqrt{1+{\left(\xi_{g}s\right)}^{2}}\right),$$ (4)

where *I* represents the Bragg peak intensity, *l* is the thickness of the crystal, $\xi_{g}$ is the extinction distance, and *s* is the parameter describing the deviation from the exact Bragg condition. According to the above theoretical analysis, the Bragg peak intensity *I* is influenced by the crystal thickness *l*, the extinction distance $\;\xi_{g}\;$, and the deviation parameter *s*. As a result, the intensity and distribution of the Bragg spot can occur over a limited range even though the Bragg condition is not exactly satisfied in most of the reciprocal lattice rods. Figure 5(a) shows a sketch of the crystal dynamic diffraction mechanism that facilitates the analysis of diffraction intensity. This diagram directly illustrates the relationships among the Ewald sphere, the reciprocal lattice rods, and the diffraction intensity of the Bragg peaks. The intersection positions of the Ewald sphere and the reciprocal rods give rise to the value of the parameter *s*—the deviation from the Bragg diffraction condition.

Diffraction intensity *I* varies in a complicated way, depending on the deviation parameter *s*, as shown in the right panel of Fig. 5(a). Our further study and data analysis reveal that the temporal evolution of Bragg peak intensity is fundamentally attributable to the Debye-Waller effect together with the reciprocal plane tilt that is caused by the breathing oscillation of the film along the c-axis during ultrafast diffraction. For instance, the larger lattice expansion following the coherent breathing oscillation along the c-axis would yield a slight tilt of the reciprocal-lattice rods, as shown in Fig. 5(b), and so the position of intersection between the Ewald sphere and the reciprocal-lattice rod could change substantially following the excitation of coherent lattice oscillation along the c-axis. In Fig. 5(b), we also illustrate the notable features of the time-resolved electron diffraction along the [212] zone axis direction. The 48 ps lattice oscillation mentioned above is considered in our analysis. So, it is clear that the reciprocal plane gradually tilts upward during the first half of each period, to t = 24 ps, and then tilts downward during the second half of each oscillation period.

It is well known that the deviation parameter *s* is one of the most important factors for understanding the fundamental diffraction properties. The deviation parameter *s* can be obtained from the deviation angle Δ*θ* as follows:^43^

$$s=\frac{\Delta \theta }{d_{hkl}}\approx \frac{\left(\delta \theta_{in}+\Delta \theta_{s}\right)}{d_{hkl}}.$$ (5)

The change in *s* after excitation by the fs-laser is induced by the lattice expansion following the coherent breathing oscillation along the c-axis. When the sample is excited by the fs-laser, Δ*θ* becomes δ*θ_in_* + Δ*θ_s_*, as shown in Fig. 5(a), and the value of Δ*θ_s_* can be expressed as

$$\Delta \theta_{s}\approx \Delta \frac{1}{d_{hkl}}/\frac{2}{\lambda }\propto \Delta d,$$ (6)

s∝Δd∝Δc, (7)

where λ is the electron wavelength. Therefore, we can define *s* = kΔc + *s*_0_ to analyze the dynamic transition in the 1*T*-TaSeTe crystal, where k is a coefficient (in our experimental fitting, k is estimated to be about 1/3(1/nm)), and *s*_0_ is the initial deviation parameter for each Bragg peak at time zero. When the excitation of coherent lattice oscillation couples with the deviation parameters *s* of different diffraction spots, the Bragg spot intensity shows remarkable temporal features. Figure 5(c) shows the evolution of intensity for three typical Bragg spots of H = -g, 3 g, and 4 g as simulated by Eq. (4) with *s*_0_ = −0.013/nm, *s*_0_ = 0.001/nm, and *s*_0_ = 0.003/nm, respectively, clearly illustrating the appearance of the two out-of-phase oscillations and a half-period oscillation arising from the excited coherent breathing mode coupled with different initial values for the deviation parameter. The values of *s* and relative intensity curves shown in Fig. 5(c) are used for illustrating the tendency of different initial *s* values. Further analysis suggests that if the intensity *I* changes monotonically during the variation of deviation parameter *s* originating from the excited coherent lattice oscillation, the intensity oscillation curve will have only a single period, and the phase of oscillation will be determined by the reciprocal space tilting direction. The multiperiod oscillations arise fundamentally from the complex correlation between the intensity and the deviation parameter *s*, as clearly shown in Fig. 5(a). This generally occurs when the initial *s*_0_ is close to the center position of the *I-s* curve. A detailed theoretical simulation of ultrafast dynamic diffraction will be reported in an upcoming paper.

Taking the Debye-Waller effect into consideration, Equation (4) can be extended to

$$I_{hkl}=\frac{1}{1+{\left(\xi_{g}s\right)}^{2}}{\text{sin}}^{2}\left(\frac{\pi l}{\xi_{g}}\sqrt{1+{\left(\xi_{g}s\right)}^{2}}\right)\times \left[\left(1-\beta \right)+\beta \;\text{exp}\left(-t/\tau_{e-ph}\right)\right].$$ (8)

**FIG. 6.**
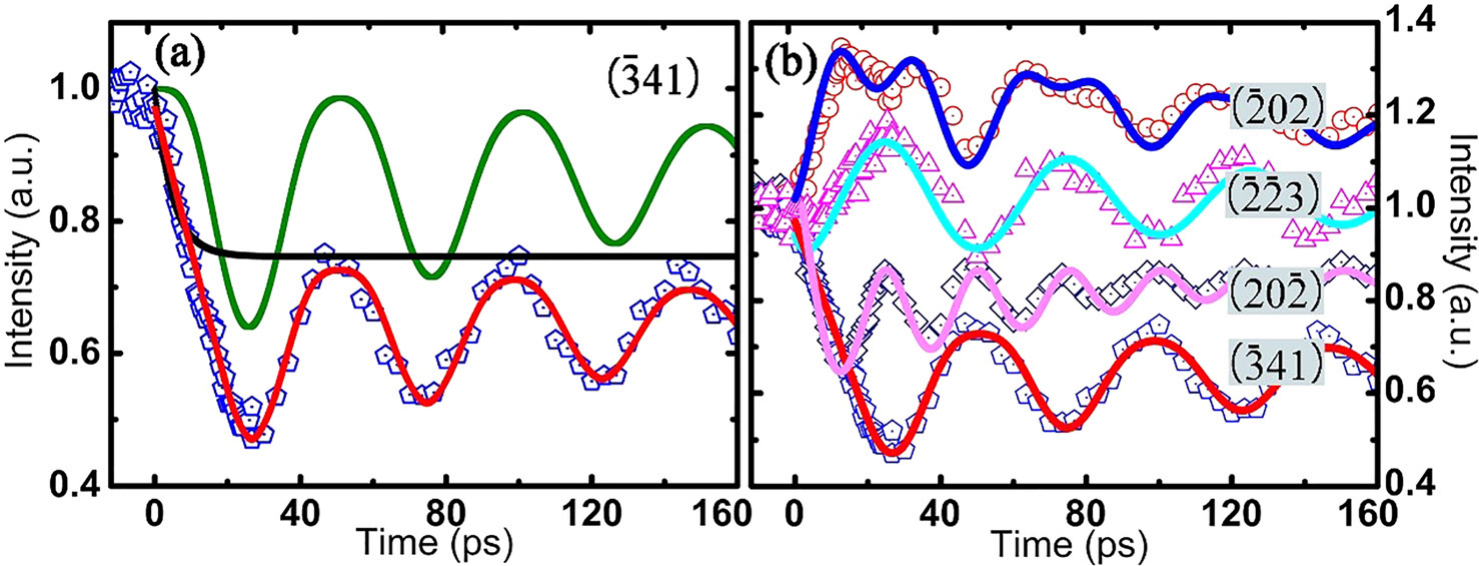
The experimental data fitted by theoretical simulations based on the dynamic diffraction theory. (a) The green curve is the intensity evolution when *s* = kΔc + *s*_0(-341)_, as shown for the –g Bragg peak in Fig. 5(c). The black solid curve is a simulation to the Debye-Waller effect. The blue pentagon is (-341) experimental data with the theoretically simulated one (the red curve). (b) The time evolution of the (-202) peak intensity can be well fitted by *s*_0_ = 0, showing a half periodicity in the lattice expansion following the coherent breathing oscillation. The experimental data for other spots can also be qualitatively simulated by *s*_0_ = 0.001/nm for (20-2), *s*_0_ = −0.013/nm for (-341), and *s*_0_ = 0.003/nm for (-2-23).

This formula is used to study the ultrafast evolution of Bragg peak intensity. Qualitatively, theoretical studies on the experimental data clearly show that the (-341) Bragg peak has a similar nature to that discussed above for the H = -g spot. In Fig. 6(a), we selected the (-341) Bragg Peak and its related extracted intensity evolution curve to explain the structural evolution in detail. The diffraction intensity for the (-341) spot can be well simulated by taking *s* = kΔc + *s*_0(-341)_ in the dynamical diffraction equation (8). The dynamical diffraction intensity oscillation, the Debye-Waller effect, the raw experimental data of the (-341) peak, and the fitted curve are all clearly shown in Fig. 6(a), showing that these theoretical data are in good agreement with the experimental data. As shown in Fig. 6(b), all our experimental data can be very well simulated by adopting the relevant *s*_0_ in Equation (8). Moreover, the (-202) Bragg Peak evolution can be well simulated by the *s*_0_ = 0 curve, showing a lattice evolution modulated by a half period of oscillations. The other curves are qualitatively simulated by *s*_0_ = 0.001/nm for (20-2), *s*_0_ = −0.013/nm for (-341), and *s*_0_ = 0.003/nm for (-2-23).

## IV. CONCLUSION

In conclusion, we have investigated the ultrafast dynamics of a two-dimensional layered 1*T*-TaSeTe material, and a rich variety of structural phenomena correlated with the lattice relaxation were extracted from the time-resolved experimental data. Our theoretical analysis based on the dynamic diffraction theory clearly demonstrates that these structural features observed in the Bragg peak evolution are attributable to the joint effects of the Debye-Waller effect and the change in the deviation parameter *s* induced by anisotropic lattice oscillation. Moreover, the intensity of the CDW satellite spots was found to be suppressed immediately following the fs-laser excitation, in good agreement with the published data. These results provide particularly convincing proof that 4D-UTEM is a comprehensive approach for exploring multiple dynamics in functional materials. In addition, this work provides a reference for the study of ultrafast diffraction kinematics and dynamics.

## V. SUPPLEMENTARY MATERIAL

See supplementary material for the complete information about the studied 1*T*-TaSeTe sample.
